# Exploring the Ecological Coherence between the Spatial and Temporal Patterns of Bacterioplankton in Boreal Lakes

**DOI:** 10.3389/fmicb.2017.00636

**Published:** 2017-04-21

**Authors:** Juan Pablo Niño-García, Clara Ruiz-González, Paul A. del Giorgio

**Affiliations:** ^1^Groupe de Recherche Interuniversitaire en Limnologie et en Environnement Aquatique, Département des Sciences Biologiques, Université du Québec à Montréal, MontréalQC, Canada; ^2^Escuela de Microbiología, Universidad de AntioquiaMedellín, Colombia; ^3^Institut de Ciències del Mar – Consejo Superior de Investigaciones CientíficasBarcelona, Spain

**Keywords:** aquatic bacterial communities, boreal lakes, abundance distributions, temporal-spatial relationship, temporal recruitment, mass effects, environmental sorting, rare biosphere

## Abstract

One of the major contemporary challenges in microbial ecology has been to discriminate the reactive core from the random, unreactive components of bacterial communities. In previous work we used the spatial abundance distributions of bacterioplankton across boreal lakes of Québec to group taxa into four distinct categories that reflect either hydrology-mediated dispersal along the aquatic network or environmental selection mechanisms within lakes. Here, we test whether this categorization derived from the spatial distribution of taxa is maintained over time, by analyzing the temporal dynamics of the operational taxonomic units (OTUs) within those spatially derived categories along an annual cycle in the oligotrophic lake Croche (Québec, Canada), and assessing the coherence in the patterns of abundance, occurrence, and environmental range of these OTUs over space and time. We report that the temporal dynamics of most taxa within a single lake are largely coherent with those derived from their spatial distribution over large spatial scales, suggesting that these properties must be intrinsic of particular taxa. We also identified a set of rare taxa cataloged as having a random occupancy based on their spatial distribution, but which showed clear seasonality and abundance peaks along the year, yet these comprised a very small fraction of the total rare OTUs. We conclude that the presence of most rare bacterioplankton taxa in boreal lakes is random, since both their temporal and spatial dynamics suggest links to passive downstream transport and persistence in freshwater networks, rather than environmental selection.

## Introduction

It is now well-established that most aquatic bacterial communities show a recurrent structure in which a relatively small number of abundant bacteria coexist with a vast number of extremely rare taxa that comprise the long tail of the so-called “rare biosphere” (Pedrós-Alió, 2006, 2012; Sogin et al., 2006). It is clear that the most abundant taxa, which are generally dynamic over time and space, are reactive to local environmental conditions within a given ecosystem, and that these taxa play an active role in the community (Fierer and Jackson, 2006; Fierer et al., 2007; Van der Gucht et al., 2007; Campbell et al., 2011; Nemergut et al., 2011; Jones et al., 2012; Shade et al., 2013). However, identifying taxa that are environmentally selected and thus likely to play an active role becomes increasingly difficult as we move deeper into the “rare biosphere,” which presumably also includes taxa with different life strategies, adaptations, preferences, and sources (Jones and Lennon, 2010; Campbell et al., 2011; Lynch and Neufeld, 2015). For example, there may be rare bacteria that are part of a microbial “seed bank” and can activate to reach high abundances under specific environmental conditions (Lennon and Jones, 2011; Gibbons et al., 2013; Aanderud et al., 2015; Baltar et al., 2015; Wilhelm et al., 2015; Ruiz-González et al., 2017), whereas other rare taxa may have intrinsically low growth and activity rates and for which rarity is a strategy (Campbell et al., 2011; Nelson and Carlson, 2012; Pedrós-Alió, 2012; Vergin et al., 2013; Logares et al., 2015). Importantly, there are taxa that are rare simply as a result of random transport to the ecosystem but cannot thrive in it, and are thus non-reactive to local environmental conditions (Hugoni et al., 2013; Vergin et al., 2013; Niño-García et al., 2016a).

As the depth of sequencing (and thus the number of taxa that are detected in any given community) increases, discriminating between taxa that are selected and thus potentially active within the ecosystem, versus those whose presence reflects dispersal and random arrival becomes an ever more complex and pressing challenge (Newton and Shade, 2016). Most of the recent research addressing the issue of activity within aquatic microbial communities, and particularly within the rare biosphere, has focused on the analysis of ratios of 16S rRNA to rRNA genes (rDNA) of individual bacterial taxa, the application of probe-based cell analysis or stable-isotope probing within a limited amount of samples or in the exploration of temporal patterns of specific microbial populations (e.g., Campbell et al., 2011; Hugoni et al., 2013; Vergin et al., 2013; Alonso-Sáez, 2014; Aanderud et al., 2015; Alonso-Sáez et al., 2015). These studies have provided fundamental insight into the distribution of activity and population dynamics within specific bacterial communities, but they do not provide a broad perspective on how taxa reactive and non-reactive to environmental conditions are distributed across aquatic ecosystems, particularly within the rare biosphere.

In a previous study (Niño-García et al., 2016b) we addressed this challenge by using the spatial abundance distributions (SpADs) of individual bacterial taxa across a wide range of boreal lakes to infer whether the presence of these taxa is the result of active selection or random transport. We statistically modeled the SpADs of individual operational taxonomic units (OTUs), and were able to cluster all the, OTUs that were detected across all lakes (118,717 OTUs) into four major categories, which we named after the model best explaining their abundance distribution (i.e., normal-like, bimodal, logistic, and lognormal). This spatial categories were further associated to distinct ecological features, such as ranges of abundances, occupancy, niche breadth, and OTUs spatial turnover between communities (Niño-García et al., 2016b). Moreover, since freshwater bacterioplankton communities are known to be highly influenced by the immigration of taxa from upstream terrestrial or aquatic ecosystems (Crump et al., 2012; Ruiz-González et al., 2015; Savio et al., 2015; Niño-García et al., 2016a), we further explored the dynamics of the resulting categories by retracing these OTUs to the fluvial networks associated to the lakes, to distinguish between taxa whose presence and abundance is driven by downstream transport versus those subjected to environmental selection within lakes. Interestingly, two of these SpAD categories (normal-like and bimodal) seemed to comprise the core of bacterioplankton communities, which included taxa whose patterns of spatial distribution were clearly linked to active growth within lakes, and to selection by local conditions (i.e., reactive taxa). The other two categories (logistic and lognormal) grouped mostly rare bacteria whose presence was linked to downstream-mediated dispersal rather than in-lake selection and growth (i.e., non-reactive), and that were therefore categorized as “random” within lakes. Although we did identify a small group of rare bacteria that nevertheless seemed to respond to local conditions in lakes, our overall conclusion was that the vast majority of OTUs (ca. 90%), representing ca. 38% of all sequences found in boreal lakes, seem to be “random” (Niño-García et al., 2016b), and thus unlikely to be playing a significant role in the biogeochemical and trophic functioning of these ecosystems.

Since these categories were based on the SpADs of individual taxa across many lakes sampled on a single occasion (mid-summer), we do not know the extent to which the ecological significance attributed to such categories is maintained over time. For example, it could be possible that the distribution of the OTUs that we found to be highly abundant and ubiquitous across space was restricted to summer conditions, and that on a temporal basis, they would be occasional taxa (*sensu* Magurran and Henderson, 2003). Similarly, rare taxa classed as “random” based on their SpADs may in fact show active recruitment and abundance peaks at other times of the year (Shade et al., 2014; Shade and Gilbert, 2015). Should that be the case, it would imply that the spatial categorization does not capture the full spectrum of bacterial functional categories in lakes, and would require incorporating a temporal dimension to effectively discriminate between truly “random” versus core lake taxa. Conversely, if these SpADs are consistent at the temporal scale, the features that they reflect should be intrinsic properties of taxa.

Studies with non-microbial organisms have shown that the rate of species accumulation in a given ecosystem results from the influence of both temporal and spatial components, suggesting that processes related with these two components influence local community assembly and scaling relationships of diversity with area and time (Adler and Lauenroth, 2003; Adler et al., 2005) but only a handful of studies have explored the interaction between species abundances over time and space (Hanski and Gyllenberg, 1993; Guo et al., 2000; McGeoch and Gaston, 2002). Interestingly, examination of the sparse evidence available suggests that infrequent species tend to have low abundances when they do occur whereas the most abundant are also more frequently found over time (Magurran, 2007), yet we do not know whether this is the case for microbial taxa. Although, several microbial studies have shown that communities change in time and space following environmental gradients (see Jones et al., 2012), to our knowledge no study has addressed whether patterns of spatial abundance of bacterial taxa are also maintained over time and vice versa, and this regardless of their environmental preferences. Exploring the spatial-temporal coherence of different categories of bacterial SpADs should provide insight not only on how the temporal and spatial dimensions will influence the structuring of microbial communities but also about the mechanisms generating rarity and commonness in natural bacterial communities.

To explore this issue, we investigated the temporal dynamics of OTUs that had been previously grouped within four categories of SpADs (Niño-García et al., 2016b) along an annual cycle in oligotrophic lake Croche (Québec, Canada), and we assessed whether there is coherence between the ecological features (abundance, occurrence, environmental ranges, and OTU turnover between communities) of the OTUs within each category over space and time. In order to extend these results beyond lake Croche, we further compared the spatial and temporal patterns of taxa across 21 additional boreal lakes for which we had data for three seasons (spring, summer, and fall). Overall, our results suggest that the ecological features associated to categories derived from the large scale spatial distribution patterns of taxa are overwhelmingly maintained over time, and thus that the core component of these bacterial communities is consistent over time and space, at least in this boreal region.

## Materials and Methods

### Study Sites, Sampling Design, and Bacterial Community Composition

In this study we present the results of a seasonal survey carried out in lake Croche, located in the Laurentian region of Québec (47°24′ N 71°47′ W), where water samples were collected monthly from July 2012 to October 2013 for the characterization of bacterioplankton communities inhabiting the epilimnion. Lake Croche is a small (18.1 ha) and moderately deep (mean depth 6 m) oligotrophic headwater lake surrounded by a pristine watershed. The lake thermally stratifies from late June through September, and is covered with ice from early October to May. The mean annual surface water temperature is 15°C, ranging from 2°C in January to 24°C in July, with a mean water retention time of 1.1 year (Carignan et al., 2000; Vachon and del Giorgio, 2014).

In addition, during 2012 and 2013, we sampled 21 lakes from four different boreal regions in Quebec (Abitibi, Bay James, Chibougamau, Saguenay), each on three occasions (spring, summer, and fall, *n* = 63). Although these lakes represent just a subset of the 198 lakes used in our previous spatial surveys (Niño-García et al., 2016a,b), with this design we were able to capture reasonably well the variation in most limnological, environmental, and hydrological characteristics (Supplementary Table 1).

To characterize bacterial communities, DNA was extracted from 0.22 μm pore-size filters after filtering 300–500 ml of water using the MoBio PowerWater DNA extraction kit, following the manufacturer’s specifications. We used the 515F and 806R primers to flank the variable regions V3–V4 of the 16S ribosomal RNA gene, which we sequenced using Illumina MiSeq2000 using a paired-end approach (Caporaso et al., 2012). We then assembled the paired-end reads by using FLASH (Magoč and Salzberg, 2011) and we obtained sequences with 250–290 bp, that were used for the downstream analysis. We removed primers and low quality, archaeal and chloroplast reads in QIIME (Caporaso et al., 2010) and we eliminated chimeric sequences using UCHIME (Edgar et al., 2011). To cluster all the sequences in OTUs without using any arbitrary similarity cutoff, we used SWARM 2.0 algorithm (Mahé et al., 2014), applying a local clustering threshold of *d* = 1, and the fastidious option to reduce the number of singletons and doubletons by grafting them onto more abundant clusters (Mahé et al., 2015). We further assigned taxonomy to the representative SWARMs by using SILVA 111 reference database (Quasti et al., 2012) and the RDP classifier (Wang et al., 2007). We finally discarded all the OTUs that were represented by <10 sequences and/or present in <10 samples and we randomly resampled the OTU table to generate an equal number of sequences per sample, based on the sample with the lowest number of reads (31,078 reads/sample in the Croche dataset and 30,900 reads/sample in the 21 lakes dataset). The objective of our sequence filtering process was to minimize the number of sequencing and processing artifacts. The sequences have been deposited in the European Nucleotide Archive, under the accession number PRJEB14062.

### Clustering Bacterial OTUs into Categories of SpADs

In this study we have assessed the temporal dynamics of bacterial taxa that had been previously categorized based on their SpADs across boreal lakes (Niño-García et al., 2016b). Details of the approach used to group bacterial taxa into categories of SpADs are presented in Niño-García et al. (2016b). Briefly, we fit the abundance distribution of individual OTUs across the 198 lakes to statistical models using the Hartigans’ dip test statistic (R Core Team, 2013; Maechler, 2015) and maximum likelihood estimation (fitdistrplus, Delignette-Muller and Dutang, 2014) and for each OTU we selected the statistical model with the lowest AIC value. This resulted in four different types of abundance distribution among all lake OTUs (normal-like, bimodal, logistic, and lognormal), and thus we grouped all OTUs with a similar SpADs together into four different broad categories. To characterize the ecological features of the different categories of SpADs we compared their ranges in mean spatial abundance, occupancy, and environmental range of their constituent OTUs. In addition, to further distinguish between categories influenced by hydrology-mediated dispersal or by environmental selection in lakes, we explored the dynamics of these OTUs across 188 additional streams and rivers (Strahler order 0 to 8) within the same boreal regions. This exercise demonstrated that normal-like and bimodal categories seemed to comprise the functional core of lake taxa, harboring bacteria that are responsive to local conditions. Conversely, logistic and lognormal categories included mostly rare taxa whose presence seemed to be associated to dispersal from the watershed (Niño-García et al., 2016b). For this study, we rebuilt the OTU table combining the 198 summer lake samples of our previous study (Niño-García et al., 2016b) together with the new sequences collected from the annual series in Croche + spring and fall in the 21 lakes. Thus, the OTU names were common and we could therefore identify which of the OTUs from lake Croche or the 21 lakes had been spatially categorized as normal-like, bimodal, logistic or lognormal, and we grouped them as such.

### Temporal Dynamics of SpAD Categories of Bacteria along the Annual Cycle in Lake Croche

We focus the analysis on OTUs detected in Lake Croche throughout the annual cycle that were also present in our previous large-scale study across lakes, and which had therefore already been classed into one of the four major categories of SpADs (Niño-García et al., 2016b). We explored the temporal dynamics in abundance of each of these OTUs along the annual cycle in lake Croche. For each OTU within the four categories, we calculated the mean OTU abundance, temporal occurrence, and environmental range, over the annual cycle in lake Croche, and we used these to derive a “temporal” average. The environmental range was calculated as the temperature range at which a particular OTU was detected because temperature was the variable showing the most pronounced changes over time, and we used it as a proxy of the seasonal variability in lake conditions. In addition, we calculated the temporal turnover of OTUs between communities (see below). The four properties calculated from lake Croche OTUs were compared with those previously calculated spatially for the four categories of SpADs across lakes (see below).

### Exploring the Presence of Temporal Shifters in Lake Croche over the Annual Cycle

We explicitly assessed whether rare taxa belonging to the lognormal and logistic spatial categories, which had been classified as spatially “random” based on their SpADs, showed temporal recruitment along the annual cycle. We identified the OTUs that were categorized as logistic or lognormal in the spatial dataset, but that showed disproportionate changes in temporal abundance along the annual cycle in lake Croche. As these classes were characterized as rare in our spatial dataset, we consider an OTU to shift in abundance when it crossed a 0.1% threshold of abundance (equivalent to 31 sequences at our rarefaction level) at least once during the whole annual cycle. Two different thresholds have been commonly used to separate abundant from rare taxa (0.1 and 0.01%, Lynch and Neufeld, 2015), here we have used the more conservative value (0.1%) to identify the rare OTUs that were nevertheless reactive over time. Out of all the OTUs that were identified as reactive over time, we further identified those whose mean spatial and temporal abundances were decoupled (i.e., such that they had systematically low spatial abundance, but attained significantly higher abundance over time in lake Croche). We refer to these OTUs as “temporal shifters.”

### Extending the Temporal Analysis to Boreal Lakes

To expand this analysis beyond lake Croche, we explored the temporal abundance patterns of the taxa identified as “temporal shifters” across the 21 additional boreal lakes, which were part of the large-scale spatial summer study but for which we had data for two additional seasons (spring and fall). As in the analysis for lake Croche detailed above, OTUs were identified as ‘temporal shifters’ if they had originally been classed in the lognormal and logistic spatial categories, and if they were rare during the summer season in all the 21 lakes but surpassed the 0.1% threshold during spring and/or fall.

### Statistical Analyses

In order to assess the coherence between spatial and temporal ecological features of the different categories, we calculated the linear correlations between the corresponding mean annual abundance, temporal occurrence, environmental range, and OTU turnover. Spatial and temporal turnover, defined as the species replacement across space and over time, were calculated based on Jaccard pair-wise dissimilarity (R betapart package, Baselga and Orme, 2012). All the analyses and figures were made in R 3.0.0 software (R Core Team, 2013).

## Results

The classification of OTUs in different categories of SpADs that we had carried out in a previous (Niño-García et al., 2016b), was only possible due to the large number of very different lakes included in the study (*n* = 198). We could not repeat the same type of analysis over time in lake Croche because the relatively small number of time points (*n* = 14) did not allow the same degree of temporal resolution. Therefore, rather than determining new categories of temporal dynamics and comparing these to the spatial categories, our approach was to identify those OTUs in lake Croche that had been previously detected in the large scale spatial analysis, and to assess whether their temporal dynamics matched the SpADs that we had previously observed for each of them. As mentioned before, this exercise was possible because the clustering of OTUs had been done using both the spatial and the temporal datasets, and thus the OTU names were common.

**FIGURE 1:**
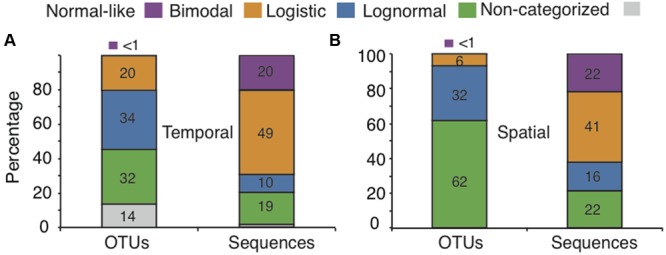
**Exploration of the coherence between the temporal and spatial ecological features for normal-like, bimodal, logistic, and lognormal categories (represented by different colors): contribution of each category to the total number of OTUs and associated sequences in lake Croche over time (A)**, and across the 198 boreal lakes **(B)**.

We obtained a total of 466,170 sequences after quality control and rarefaction for the annual cycle in lake Croche (referred to as the “temporal” dataset hereafter), which clustered into 30,708 OTUs. A large majority of the OTUs (86%) that were present in lake Croche had already been detected in the previous large-scale survey across 198 boreal lakes (Niño-García et al., 2016b), and had thus been classed into one of four categories of SpADs identified in that study. Only 4,162 OTUs (14% of Croche OTUs, ‘Non-categorized’ OTUs) were not found in the spatial dataset but these accounted for a very small proportion of the sequences within the temporal dataset (<2%, **Figure 1A**).

The majority of OTUs that we detected in lake Croche (66%) had been previously classed as either logistic or lognormal based on their SpADs, but together accounted for a relatively small proportion (29%) of all sequences detected in the lake over the annual cycle. On the other hand, most of the sequences (69%) detected in lake Croche were associated to a small proportion of OTUs (34%) that had been previously classed in the normal-like and bimodal categories based on their SpADs (**Figures 1A,B**). This overall distribution of OTUs and sequences detected over lake Croche annual cycle into the four categories of SpADs was remarkably similar to that observed over space for the ensemble of lakes, where we had found that over 94% of all OTUs found across all lakes classed into logistic and lognormal categories but accounted for relatively few sequences (38%), whereas the small number of OTUs classed into the normal and bimodal categories accounted for a large proportion (63%) of all sequences detected across lakes (**Figures 1A,B**).

**FIGURE 2:**
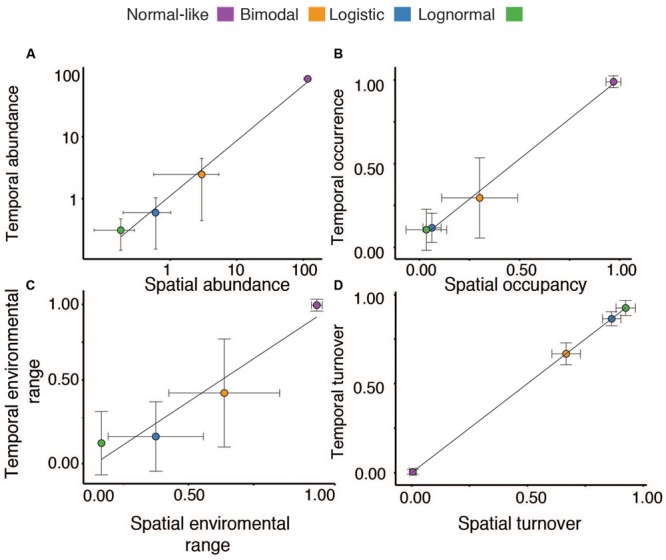
**Relationship between the average temporal (Y-axis) and spatial (X-axis) mean abundances (A)**, temporal occurrence and spatial occupancy **(B)**, environmental ranges (estimated based on temperature in the case of Croche and pH in the spatial study, see Materials and Methods) **(C)**, and OTU temporal and spatial turnover **(D)** for the different normal-like, bimodal, logistic, and lognormal (green) categories. The dots represent the mean and the error bars the standard deviation associated to each mean.

**FIGURE 3:**
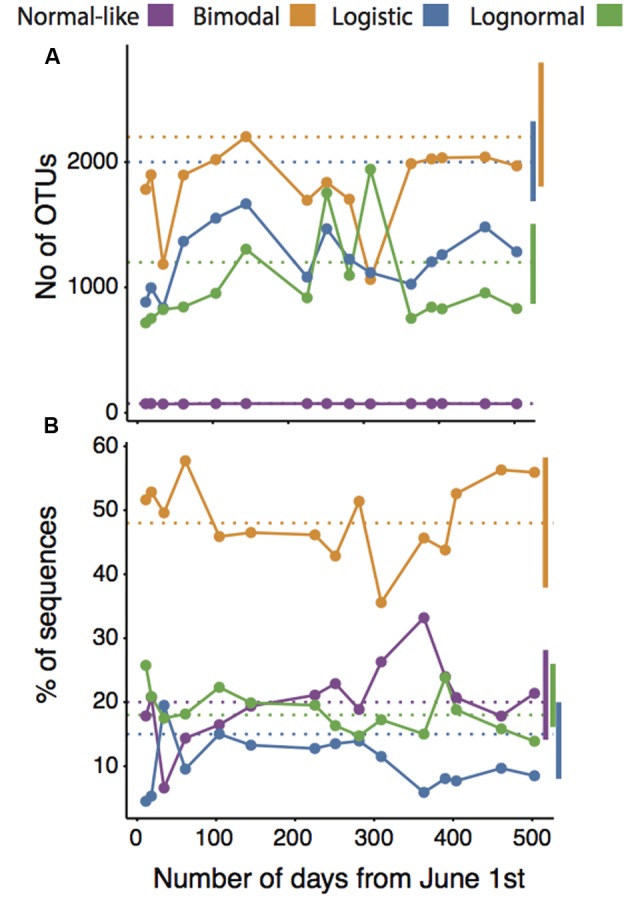
**Temporal patterns in the number of OTUs (A)** and the percentage of sequences **(B)** associated to normal-like, bimodal, logistic, and lognormal categories along the annual cycle in lake Croche, in relation to their respective means (dotted horizontal lines) and ranges of variation (vertical bars) derived from the large-scale spatial dataset (198 lakes).

There was a very strong correlation between the mean spatial and temporal OTU abundances, occupancy-occurrence, environmental ranges and the OTU turnover, suggesting a strong overall coherence between the ecological features of our four categories on temporal and spatial scales (**Figures 2A–D**). For example, OTUs that were spatially normal-like seemed to be also very abundant and ubiquitous over the annual cycle in lake Croche, whereas most logistic and lognormal OTUs based on their spatial distributions were extremely rare and present only in a few occasions annually (**Figures 2A–D**). The number of logistic OTUs seemed slightly below the range of variation in the spatial survey, yet it is possible that most of the very rare ‘Non-categorized’ OTUs (**Figure 1A**) showed a logistic pattern, in which case the numbers would agree. Similarly, although the number of OTUs associated to the bimodal and logistic categories in lake Croche was slightly below the average values in the spatial dataset (**Figure 3A**), in general the temporal variability in the number of OTUs classed within each of the four spatial categories, and the number of associated sequences, fell well within the spatial range of variation in these parameters observed across the 198 lakes (**Figures 3A,B**). These results confirm that these normal-like and bimodal OTUs that appeared to be selected by lake environmental conditions spatially and thus conforming the core of bacterial communities across the 198 lakes (Niño-García et al., 2016b), also dominated the lake Croche bacterial community over time, and which were reactive to environmental changes along an annual cycle.

**FIGURE 4:**
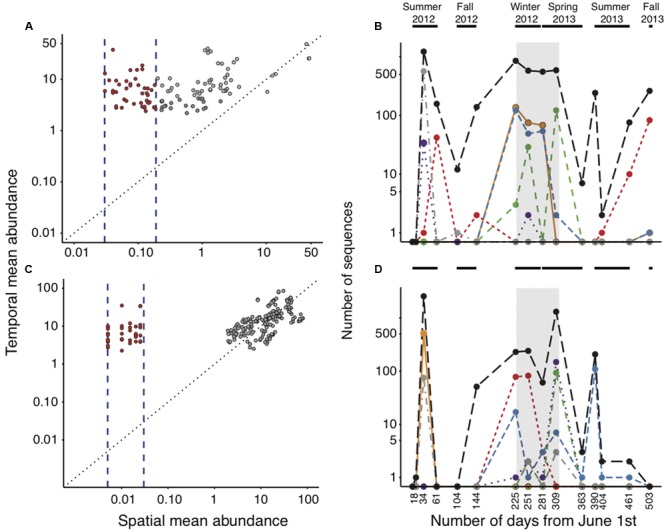
**Relationship between the spatial and temporal mean abundance of logistic (A)** and lognormal **(C)** OTUs that crossed the 0.1% abundance threshold along the annual cycle in lake Croche. Temporal shifters (red circles) are OTUs that had a disproportionately high relative abundance in lake Croche over the annual cycle (position over the 1:1 black dotted line) and a mean spatial abundance that was within the range used to define it either as logistic or lognormal (vertical blue dashed lines). Gray circles correspond OTUs discarded as temporal shifters, which were not within the ranges of typical logistic and lognormal categories (i.e., they were outliers both in the temporal and the spatial surveys). Examples of temporal dynamics of representative individual logistic **(B)** and lognormal **(D)** temporal shifters in lake Croche. Different colors and/or line types correspond to individual OTUs, and the black dashed line represents their pooled sequences. Horizontal black lines on top of the plots show the corresponding sampling seasons and the vertical shade represent the ice-covered period.

We were particularly interested in assessing whether any of the rare OTUs classed in the logistic and lognormal categories, whose spatial distribution was considered as random and hydrology-mediated (Niño-García et al., 2016b), showed active recruitment along the annual cycle in lake Croche. We found that the vast majority (ca. 99%) of these OTUs were also systematically rare in Lake Croche, never exceeding an arbitrary abundance threshold of 0.1% at any point of the annual cycle, and never showing any kind of clear seasonal dynamics, confirming that most spatially rare taxa remain temporally rare as well. We detected a small subset of spatially rare taxa, however, which did show seasonal transitions from rare to abundant (details not shown), composed of 115 logistic OTUs and 206 lognormal OTUs that exceeded the 0.1% abundance threshold at least once over the course of the annual cycle. It is interesting to note that for over half of these OTUs there was a strong relationship between their average spatial and temporal abundances, which suggests that there was coherence between their spatial and temporal patterns of distribution (gray dots in **Figures 4A,C**, which were indeed outliers both in the spatial and the temporal dataset). Only 43 and 36 OTUs, respectively, showed a clear decoupling between their respective spatial and temporal mean relative abundances (dots between the vertical lines in **Figures 4A,C**), having much higher mean temporal abundances within lake Croche than their average summer abundance across all lakes. These temporal shifters thus represent OTUs that were spatially rare during the summer across all lakes, but that recruited to higher abundances at other times of the year in lake Croche. Although these shifter OTUs had peaks at different times of the year (see examples of individual OTUs in **Figures 4B,D**, their aggregated dynamics (black line in **Figures 4B,D**) showed a higher prevalence of such shifts in abundance in times of the year other than summer (**Figures 4B,D**). It is clear that this group of temporally shifting OTUs is not well-categorized solely on the basis of their spatial dynamics across lakes in summer, yet it is important to note that they comprise a negligible fraction of the total number of OTUs identified within the logistic and lognormal rare categories (0.5 and 0.7%, respectively). Together, these results demonstrate that only a very small proportion of the spatially rare, seemingly random OTUs (i.e., logistic and lognormal) appear to recruit temporally and ever become part of the core component of the community in lake Croche.

In order to assess the generality of these findings, we explored the distribution and prevalence of temporal lake Croche shifters in an additional dataset of 21 lakes for which we had samples for three distinct seasons (summer -also included in the spatial dataset-, spring and fall). Of the 79 lognormal or logistic temporal shifters previously identified in Lake Croche (**Figures 4A,C**), 72 were also present in at least one of the 21 lakes (mean per lake = 4, range: 0–21) but they were all consistently below the 0.1% abundance threshold in all three seasons, and therefore did not behave as temporal shifters in these lakes. This suggests that the logistic and lognormal taxa that shift temporally may be highly lake-specific, as opposed to the normal and bimodal taxa, which are widespread across all lakes, and also in time within a given lake.

In order to further test this hypothesis, we independently identified temporal shifters in the 21-lake dataset by selecting the OTUs whose abundance was below the 0.1% abundance threshold in summer (when their spatial categorization was made in Niño-García et al., 2016b), but which became abundant in spring and/or fall. This procedure recovered a total of 127 shifter taxa belonging to the logistic and lognormal categories (**Table 1**). Interestingly, the mean number of shifter OTUs per lake was similar to that found in lake Croche (**Table 1**), suggesting that in lakes in general there may exist a small and rather constant pool of taxa that appear to be spatially rare, but which spike at very specific times of the year, and which are therefore reactive to certain lake environmental conditions.

**Table 1:** Number of operational taxonomic units (OTUs) and percentage of sequences categorized as temporal shifters in lake Croche and in the dataset of 21 lakes for which we had seasonal data (see text).

		Number of OTUs	% of sequences
Lake Croche	Total	79	–
	Logistic	43	0.01
	Lognormal	36	0.01
Logistic in 21 lakes	Total	60	–
dataset	Mean	13	0.03
	Range	0–33	0–2
Lognormal in 21 lakes	Total	67	–
dataset	Mean	25	0.2
	Range	8–45	0.1–3.5

## Discussion

Modern sequencing approaches have revealed that aquatic and terrestrial bacterial communities are composed of 1000s of taxa, the vast majority of which are extremely rare, both in abundance and in their patterns of occupancy across systems (Curtis et al., 2002; Sloan et al., 2006; Lynch and Neufeld, 2015). The structure of these communities results from a combination of active growth within the ecosystem, dispersal from outside the system (Pedrós-Alió, 2006, 2012), and passive persistence (Newton and Shade, 2016), but one of the major contemporary challenges in microbial ecology has been to discriminate the core from the random components of bacterial communities (Caporaso et al., 2011; Staley et al., 2014; Saunders et al., 2015). In a companion paper we addressed this fundamental issue explicitly for lake bacterial communities (Niño-García et al., 2016b), known to be strongly structured by both environmental selection and immigration from adjacent ecosystems (Crump et al., 2012; Ruiz-González et al., 2015; Niño-García et al., 2016a). In that previous study we had determined the spatial abundance distribution (SpAD) of individual bacterial OTUs (determined on the basis of deep sequencing of 16S rDNA) across 198 widely different boreal lakes in Québec, and we used these to group taxa into categories of SpADs. This partition of the structure of lake bacterial communities into four groups of taxa with different SpADs suggested that lake communities are composed of a core of taxa whose distribution is linked to active growth and in-lake environmental selection (normal-like and bimodal categories of SpAD), and an enormous fraction of rare bacteria (94% total OTUs) whose presence seems random and linked to hydrology-mediated transport (logistic and lognormal categories, Niño-García et al., 2016b).

A major outstanding question that emerged from the above studies is whether this functional discrimination of lake bacterial taxa based on their mid-summer spatial distribution across boreal lakes may have a temporal dimension, wherein for example taxa that are spatially rare and apparently “random” may in fact be reactive at other times of the year, or taxa that appear to be widespread and consistently abundant disappear in particular seasons. In this study we explicitly addressed this question, by assessing the temporal dynamics of those taxa that had been previously classified on the basis of their large-scale SpADs across lakes. The number of temporal samples that we collected from lake Croche did not allow us to model the abundance/occurrence distributions over time as we had done across lakes (14 temporal samples compared to 198 spatial samples), so we based our analysis on identifying OTUs in lake Croche that had previously been identified in the 198 lakes and classified into one of the four categories of SpAD. This allowed us to study whether the ecological features attributed to these taxa based on their spatial dynamics do apply also when the temporal scale is considered, and therefore, the extent to which this spatial categorization of taxa reflects intrinsic properties or capacities of individual bacterial OTUs. Our results demonstrate a high degree of coherence between the ecological features inferred from the patterns of spatial and temporal distribution of bacterial taxa, in particular concerning the distinction between taxa that are reactive to environmental conditions versus those that are non-reactive and whose presence in lakes appears to be random (**Figures 1, 2**).

In our previous study, we had established that all lake communities contain a small group of normal-like OTUs that are both consistently abundant and widespread across lakes, which appear to be highly tolerant to wide environmental gradients (Niño-García et al., 2016b). Here, we show that most of those OTUs that were previously categorized as normal-like were also present in lake Croche year round, and accounted for a large fraction of the total sequences detected throughout the annual cycle (**Figures 1, 2, 3B**). The temporal dynamics of this group as a whole OTUs supports the previous conclusion based on their SpADs that this category represents ubiquitous taxa possibly with high intrinsic growth rates that are tolerant to wide environmental ranges (Mou et al., 2008; Newton et al., 2011; Lennon et al., 2012; Székely and Langenheder, 2014), and therefore present not only across all lakes but also throughout the year within a given lake (**Figure 3B**). The second major category of bacterial taxa that we had identified from their SpADs were the bimodals, composed of OTUs with much more variable abundances and occupancy across lakes than the OTUs within the normal-like category, and in general with a narrower range of environmental preference, yet collectively accounting for the largest proportion of the sequences in the spatial dataset (Niño-García et al., 2016b, **Figure 1B**). These features were essentially replicated in time over the annual cycle in lake Croche (**Figure 1A**), and OTUs belonging to the spatial bimodal category had strong seasonal fluctuations, as reflected by their high temporal turnover, and which collectively accounted for a large fraction of the total number of sequences detected in the lake (**Figures 2A,C, 3B**), as was the case across the 198 lakes. Thus, whereas normal-like OTUs are consistently present and abundant in lake bacterioplankton communities over space and time, bimodal OTUs represent bacteria that, while also reactive to lake conditions, tend to cycle between the rare and abundant fractions of the community possibly due to their narrower range of preferences and/or tolerances (Lennon et al., 2012; Salcher, 2013; Lynch and Neufeld, 2015; Shade and Gilbert, 2015). In any case, the temporal and spatial patterns converge to suggest that these two categories likely harbor the bulk of the active component of lake bacterioplankton communities, thus likely representing the functional core of these communities as proposed by Shade and Handelsman (2012) and Saunders et al. (2015).

In our previous study, the vast majority of the rare taxa fell into two distinct categories of SpADs (logistic and lognormal) based on their cross-lake changes in abundance, and their patterns along the associated fluvial networks suggested that their presence in lakes was mostly driven by dispersal from the watershed (Niño-García et al., 2016b). We had thus hypothesized that most of these taxa are random within lakes, likely due to a combination of downstream transport, persistence and dilution. In this regard, we expected that the temporal patterns of these “random” taxa should be influenced by temporal variations in hydrological loading within the lake, and thus for example we would expect a higher proportion of logistic and lognormal OTUs in times of high water inputs, such as during snow melt or after strong storms. We did not detect any clear seasonal pattern associated to such events in these two spatial categories (details not shown), but their aggregated ecological features were very similar in both the spatial and temporal datasets: they represented the majority of OTUs found along the annual cycle in Lake Croche (**Figures 1, 2**), yet accounted for fewer than half of the total sequences (**Figure 3B**), had very low average abundance and an extremely patchy temporal occurrence, a pattern that is very similar to what we had previously found spatially across lakes (Niño-García et al., 2016b). There was therefore an overall tight coherence between the spatial and temporal dynamics of these rare taxa as a whole, and no clear evidence that the inclusion of a temporal dimension alters the conclusion that their presence in lakes is mostly random.

It is possible, however, that individual taxa within this overall pool of rare, apparently non-reactive bacteria, may recruit at specific times of the year (Alonso-Sáez, 2014; Shade et al., 2014; Shade and Gilbert, 2015), and this might not be reflected in the average temporal dynamics of the group as a whole. Interestingly, when exploring the individual temporal patterns of all the OTUs classed in the lognormal and logistic categories, we found a very small number of OTUs (79) that were spatially rare but which became temporarily abundant in lake Croche (temporal shifters, **Figures 4A,C**). The overwhelming majority of lognormal and logistic OTUs remained below the abundance threshold we set (0.1%) and were patchily distributed throughout the annual cycle, again confirming that these taxa are mostly unreactive to lake environmental conditions (Niño-García et al., 2016b). It is interesting to note that the temporal patterns were remarkably similar among some of the temporal shifters that we identified (**Figures 4C,D**), suggesting either strong eco-physiological similarities between these taxa, or a common source. Most of these shifters tended to peak in winter, suggesting that both temporal changes in local environmental conditions and unusual loading events may underlie the temporal spikes of these taxa (**Figures 4B,D**). This dynamics would be analogous to that of some conditionally rare taxa identified by Shade et al. (2014), which have been shown to contribute disproportionately to temporal changes in microbial communities, but in lake Croche these potential shifters comprise a very small fraction of the vast pool of rare bacteria. Rather, the vast majority of the taxa that showed strong temporal variations in abundance and occurrence were spatially bimodal taxa, confirming that the reactivity of the taxa in this category to lake environmental conditions is expressed both spatially across lakes, and temporally within a given lake (**Figures 2A,B**).

The relatively small number of rare (logistic and lognormal) taxa that we identified as temporal shifters is rather surprising, and may be the result of having considered only one lake for this temporal analysis. If such pool of rare OTUs that can potentially recruit at specific times over an annual cycle were very site-specific, the size of this reactive pool of spatially rare taxa should increase progressively with an increasing number of lakes considered. However, when we repeated the analysis of the temporal patterns of individual logistic and lognormal OTUs for the additional 21 lakes for which we had seasonal data, we recovered only 1.6 fold more temporal shifters than when considering lake Croche alone (127 versus 79, **Table 1**). Although we acknowledge the limited temporal resolution of this multi-lake dataset, especially considering that many shifters in lake Croche recruited during winter (**Figures 4B,D**), it does not seem that increasing the number of lakes proportionately increased the apparent size of this pool of rare, temporally reactive bacteria. In addition, we found that whereas most of the temporal shifters that we identified in lake Croche were detected in the 21-lake dataset, these were always below the 0.1% threshold of abundance and at relative low occurrences (ca. 20%) in the 21 lakes. The spatial turnover of these temporal shifters OTU across the 21 lakes (spatial OTUs turnover 0.2–0.7) suggests an incomplete replacement of temporal shifters between lakes (i.e., many are shared between lakes), but their ability to recruit seems system-dependent because most of these shifters peaked in only one or two lakes.

We acknowledge that sequence processing and the different quality-filtering strategies and clustering methods could influence the actual number of OTUs recovered. Our approach, which combines QIIME settings and SWARM, was explicitly designed to maximize the removal of the potential artifacts, particularly on the rare component of the community (Bokulich et al., 2013; Kopylova et al., 2016) and our results in terms of the number and identities of the OTUs are very consistent with the findings of our previous work using similar datasets Thus, although the absolute OTU numbers reported on this manuscript must be interpreted with caution, we believe that the potential variation does not influence dramatically our main conclusions about the coherence between temporal and spatial distributions of bacterial taxa. We further acknowledge that the 0.1% abundance threshold that we used to identify these logistic or lognormal temporal shifters is arbitrary, although it has frequently been used in previous papers to separate rare versus abundant taxa (Pedrós-Alió, 2006; Fuhrman, 2009; Vergin et al., 2013). The use of an arbitrary abundance threshold to identify reactive taxa surely imposes a bias in our results, since in reality there is no absolute measure of rarity, but we should emphasize that it is the pattern that is of importance here, not the actual number of OTUs identified. In this regard, increasing or lowering this threshold by an order of magnitude slightly changes the numbers, but in no way changes the overall conclusion that the vast majority of rare OTUs classed within the lognormal and logistic spatial categories do not show any discernible temporal pattern in lakes. Our results thus support our previous observation that the lake “rare biosphere” is mostly composed of OTUs whose presence is accidental and linked to hydrologic dispersal (Niño-García et al., 2016b).

Overall, we show for the first time that the ecological features derived from the patterns of spatial abundance distribution of bacteria across space seem to be ecologically robust and inherent to taxa, since their dynamics over time are largely what we expected from each of the four categories of OTUs. In general, taxa that are able to grow within a very broad range of lake environmental conditions (normal-like) are also able to tolerate temporal changes in these conditions and dominate year long. Taxa that are restricted to a more limited set of environmentally similar lakes (i.e., bimodal) will also tend to be prevalent at more specific times of the year, and bacteria whose spatial patterns are the result of downstream dispersal and passive persistence (lognormal and logistic) also tend to be unreactive at the temporal scale. Interestingly, the remarkable coherence in the contribution of each of these four categories to the structure of the communities suggests that this must be a recurrent property of boreal freshwater bacterioplankton assemblages. This implies that the reactive core of lake bacterioplankton communities is mainly composed by an adaptive group of normal-like and bimodal OTUs that responds to spatial and temporal environmental gradients. In contrast, downstream-dispersal processes drive the distribution of the vast majority of random bacteria, which do appear to be reactive to ambient conditions and likely play a limited role in community processes.

Our results further support the view that biological communities can be partitioned into subsets of taxa whose different abundance distributions result either from stochastic processes related with dispersal or from niche-driven mechanisms associated to selection by local conditions that together influence the shape of the species abundance distributions (Hanski, 1982; Magurran and Henderson, 2003; Ulrich and Ollik, 2004; Ulrich and Zalewski, 2006). The large role of dispersal on structuring freshwater bacterioplankton communities and its variation across the landscape (Ruiz-González et al., 2015; Niño-García et al., 2016a) suggests that the balance between the core and the random components of bacterioplankton communities likely changes along the aquatic continuum: in fast-flowing headwater streams, which are strongly subjected to inoculation of microbes from soils (Ruiz-González et al., 2015), the contribution of such random taxa may be relatively more important than downstream in the networks, whereas the core component will make up a larger fraction of the communities in systems with higher water residence times. The coherence between the spatial and temporal properties measured here for the different categories of SpADs, together with the remarkably small number of temporal shifters, indicate that similar processes underpin spatial and temporal abundance distributions of bacterioplankton taxa in lakes, and that bacterial rarity on these ecosystems is largely the result of hydrologic mediated transport of unreactive bacterial taxa.

## Author Contributions

PdG, JN-G designed the sampling, JN-G, PdG collected the data, JN-G and CR-G analyzed the data, JN-G, PdG, and CR-G discussed and interpreted the results and JN-G, CR-G, and PdG wrote the manuscript.

## Conflict of Interest Statement

The authors declare that the research was conducted in the absence of any commercial or financial relationships that could be construed as a potential conflict of interest.
