# Supplementary material for: Exploring the Ecological Coherence between the Spatial and Temporal Patterns of Bacterioplankton in Boreal Lakes
Source: Front Microbiol. 2017 Apr 21;8:636. doi: 10.3389/fmicb.2017.00636 (PMC5399088; doi:10.3389/fmicb.2017.00636)
Supplement: Supplementary file 1 [file Table_1.DOCX]

Table Supplementary 1. Range of main limnological and landscape variables measured in the 21-lakes and 198-lakes dataset. Whereas for 198 lakes the ranges correspond to a summer snapshot, the 21-lakes dataset includes the variation in spring, summer and fall.

|  | **21-lakes dataset** | | **198-lakes dataset** | |
| --- | --- | --- | --- | --- |
| **Variable** | **Minimum** | **Maximum** | **Minimum** | **Maximum** |
| pH | 5.56 | 9.22 | 4.04 | 8.95 |
| DOC (mg/L) | 3.96 | 20.25 | 1.064 | 39.67 |
| cDOM (m^-1^) | 0.01 | 8.64 | 0.01 | 20.04 |
| TN (mg/L) | 0.4 | 0.89 | 0.1 | 0.91 |
| TP (µg/L) | 2.88 | 117.6 | 2.58 | 153 |
| TEMP (C) | 7.5 | 24.06 | 10.75 | 24.7 |
| CHL (µg/L) | 0.39 | 10.24 | 0.14 | 36.05 |
| WRT(days) | 17 | 10523 | 1.38 | 10523 |
| Zmax (m) | 0.7 | 70 | 0.5 | 78 |
| Secchi (m) | 0.1 | 6.5 | 0.1 | 9.5 |
